# Predictive factors for positive disco-vertebral biopsy culture in pyogenic vertebral osteomyelitis, and impact of fluoroscopic versus scanographic guidance

**DOI:** 10.1186/s12879-020-05223-z

**Published:** 2020-07-16

**Authors:** Caroline Diffre, Camille Jousset, Anne-Laure Roux, Clara Duran, Latifa Noussair, Martin Rottman, Robert-Yves Carlier, Aurélien Dinh

**Affiliations:** 1 Department of medical imaging, Raymond Poincaré University Hospital, AP-HP Paris Saclay University, 92380 Garches, France; 2 Microbiology laboratory, Raymond Poincaré University Hospital, AP-HP Paris Saclay University, 92380 Garches, France; 3 Infectious disease unit, Raymond Poincaré University Hospital, AP-HP Paris Saclay University, 104, boulevard Raymond Poincaré, 92380 Garches, France

**Keywords:** Vertebral osteomyelitis, Fluoroscopy, DVB, MRI, Paravertebral infiltration

## Abstract

**Background:**

The aims of this study were to identify the predictive factors for microbiological diagnosis through disco-vertebral biopsy (DVB) in patients with pyogenic vertebral osteomyelitis (PVO) and negative blood cultures, and compare the performance of DVB under fluoroscopic versus scanographic guidance.

**Methods:**

We performed a cohort study comparing positive and negative DVB among patients with PVO. All cases of PVO undergoing a DVB for microbiological diagnosis in our center were retrospectively reviewed. Infections due to *Mycobacterium tuberculosis*, infections on foreign device, and non-septic diseases were excluded. Anamnestic, clinical, biological, microbiological, as well as radiological data were collected from medical charts thanks to a standardized data set.

**Results:**

A total of 111 patients were screened; 88 patients were included. Microbiological cultures were positive in 53/88 (60.2%) patients. A thickening of the paravertebral tissue ≥10 mm on magnetic resonance imaging (MRI) in axial MR scans was a predictive factor of DVB microbiological positivity (52.4% vs. 13.3%; *p* = 0.006; OR = 5.4). Overall, 51 DVB were performed under fluoroscopic guidance and 37 under scanographic guidance. Considering lumbar DVB, 25/36 (69.4%) of cases yielded positive results under fluoroscopic guidance versus 5/15 (33.3%) under scanographic guidance (*p* = 0.02; OR = 4.4). No adverse event linked to DVB was notified.

**Conclusion:**

Every patient with PVO and negative blood cultures should undergo a DVB. A thickening of the paravertebral tissue ≥10 mm on MRI is associated with a higher rate of positive DVB culture. A lumbar DVB under fluoroscopic guidance is more sensitive than under scanographic guidance to identify the micro-organism involved.

## Background

Pyogenic vertebral osteomyelitis (PVO) is an infection of the disk and the corpus of the adjacent vertebrate, which may extend to the epidural or paravertebral area. It is usually due to blood stream dissemination (80% of cases), rather than a direct inoculation during surgery or radiological intervention, such as disco-vertebral biopsy (DVB) [1]. *Staphylococcus aureus* is the most frequent pathogen involved [2, 3].

It is a rare disease but its incidence has risen in recent years [4]. It is now estimated at 2,4 per 100,000 habitants per year, increasing with age [2]. The lumbar level is the most frequent affected part of the spine (more than 50% of cases) compared to dorsal (30%) and cervical spine (20%) [5–7]. PVOs are characterized by non-specific presentation: back pain (80%), fever (50%), or neurological symptoms in case of complication [8]. Therefore, the diagnostic delay varies between 3 and 13 weeks [8, 9]. Magnetic resonance imaging (MRI) is the more sensitive and specific technic for positive diagnosis. It allows to distinguish infections from degenerative diseases, such as Modic 1 inflammatory discitis or microcrystalline disease.

Identification of a causative microorganism is a key point in management of PVO. Indeed, it allows an effective and targeted antimicrobial treatment on the bacteria involved. Blood cultures may identify the causative organism in up to 40 to 60% of cases [10]. In patients with negative blood cultures, a percutaneous DVB is recommended [8].

DVB could be performed under fluoroscopic or scanographic guidance. However, guidelines do not specify which modality is preferential. In the literature, DVB are mainly performed under scanographic guidance, which may be due to an apparent safety, especially at the thoracic level due to proximity of the aorta [11–13].

The reported DVB’s sensitivity is around 50%, and vary from 30 to 91% [8, 13–17]. But, a study comparing the two types of guidance has never been performed to best of our knowledge.

The primary objective of our study was to identify the predictive factors of DVB positive cultures. The secondary objective was to compare the performance of DVB under fluoroscopic versus scanographic guidance.

## Methods

### Study design and patients

We performed a retrospective study of all patients who underwent DVB for PVO with negative blood cultures between January 1st, 2002 and December 31st, 2017 in a French tertiary care hospital, identified using the local register of interventional procedures.

The diagnosis of PVO was assessed by an independent committee comprised of 2 radiologists (CD1 and RYC) and one infectious disease physician expert in PVO (AD) after reviewing all available radiological exams and clinical and biological data. If all members disagree on a patient’s classification, the patient’s data was reviewed jointly during a formal meeting to find a consensus,

No patient included in the study expressed opposition to the use of clinical data in this retrospective study. Because of its retrospective design, no institutional review board approval was needed. The study was done in accordance with the ethical principles of the Declaration of Helsinki and the Guidelines for Good Clinical Practice.

The following data were collected using a standardized record data set: technical data, i.e. guidance’s modality (scopic versus fluoroscopic guidance), level of the DVB (D11-D12 was considered on thoracic level, L5-S1 on lumbar level); bacteriological data; baseline clinical data, such as age, sex, previous spine surgery without material (laminectomy, discectomy), antibiotics during the previous 21 days before DVB, disease presentation (acute: clinical-radiological diagnosis delay lower than 30 days; subacute: clinical-radiological diagnosis delay between 30 days and 6 months; chronic: clinical-radiological diagnosis delay higher than 6 months), presence or absence of fever during the 24 h following the DVB (defined as temperature higher than 38 °C); biological data; and radiological findings such as para-vertebral, discal, or psoas abscess, paravertebral infiltration more or equal to 10 mm, vertebral endplates erosions more than 50% of the height of vertebral body, and epiduritis.

### DVB

The vertebral level was defined according to pre-interventional MRI. All patients received moderate sedation using 1 g of paracetamol. Sedation and analgesia could secondarily be assured by medical nitrous oxide and oxygen gas mixture.

CT-guided biopsy was performed on a Somaton Definition AS plus (Siemens Healthcare SAS, Erlangen 91,052, Germany). A thin-slice planning CT scan (0.75 mm slice thickness) was realized in prone position and multiplanar reconstructions were used to non-traumatically position the biopsy needle.

Fluoroscopic-guided biopsy was performed on a Philips VELARA Integris ALLURA table (Philips Healthcare, Best 5684 PC, Netherlands).

Chlorhexidine-isopropylic alcohol overlying the biopsy needle trajectory and consecutive local anesthesia with 10 mL lidocaine 0,5% (Aguettant, 69,007, Lyon, France) and then with lidocaine 0,5%-chirocaine 2,5 mg/mL (Abbvie, North Chicago, USA) in touch with periostea were used for aseptic preparation of the skin.

A small skin incision was made and biopsy was performed using T’AM kit (Thiebaud Biomedical Devices, Margenciel, France) with a 13 G biopsy system, Laredo-Bard Trocar (VM-Tech, Cachan), bone marrow biopsy needle Quick-Core (Cook, Bloomington, USA).

**Fig. 1:**
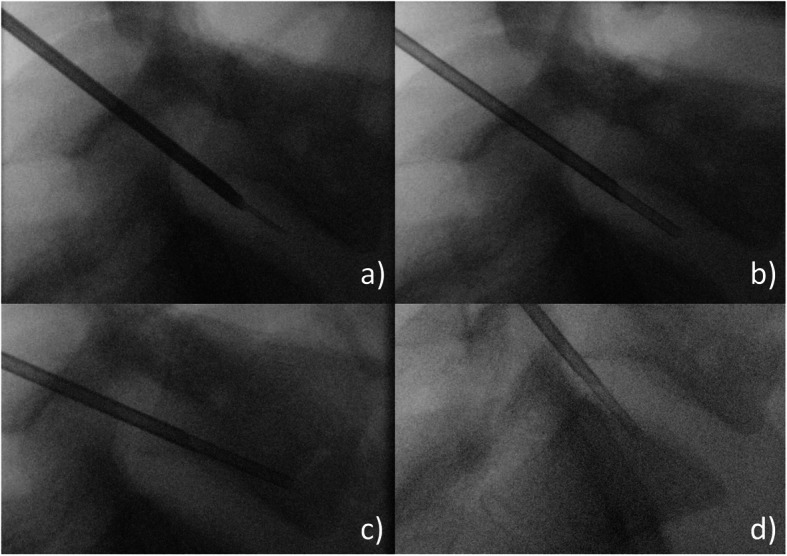
Disco-vertebral biopsy (DVB) under fluoroscopic guidance at the L5-S1 level. DVB at this level needs a large craniocaudal angulation and will target the L5 lower vertebral body rather than the L5-S1 disk or S1 upper vertebral body because bone marrow yields better samples than disk, and targeting the S1 upper body is difficult due to the overlaying shadow of the iliac bones. **a** Trocard on the guide needle. **b** Sample of L5-S1 space. **c** Sample of inferior vertebral body endplate of L5. **d** Sample of superior vertebral body endplate of S1

**Fig. 2:**
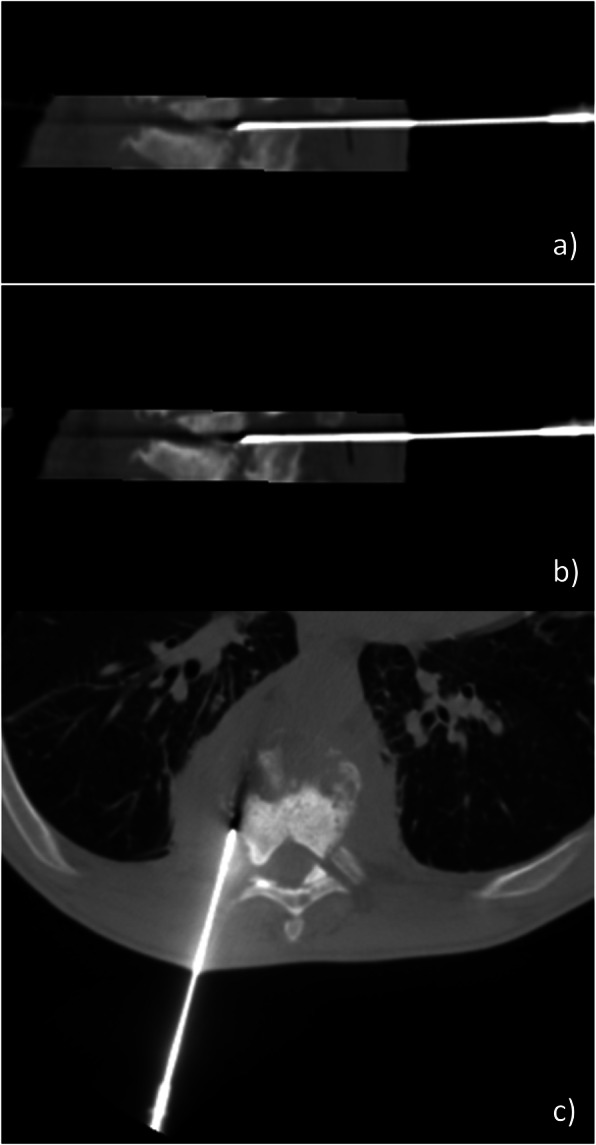
DVB under scanographic guidance at T8-T9 level. **a** Position of the needle guide at T8-T9 disk space. **b** Sample of inferior vertebral endplate of T8. **c** Inter-costo-vertebral approach

For DVB under fluoroscopic guidance, we used a postero-lateral approach with a patient in 3/4 on the table (Fig. 1) [8]. A transpedicular, inter-costo-vertebral or postero-lateral approach can be chosen for DVB under scanographic guidance (Fig. 2). According to the guidelines, several bone and discal samples were realized [8].

### Microbiology

Intraoperative samples obtained from DVB were processed independently. They were topped with 17 mL sterile distilled water and bead milled for 150 s on a Retsch MM400 mixer mill (Verder, France) with 10 to 15 5-mm-diameter stainless steel beads. One hundred microliters of the resulting suspension was plated on 5% sheep blood Columbia agar and chocolate agar and incubated for 5 days at 35 °C under aerobic, anaerobic, and 5% CO2-enriched atmospheres. Enrichment was performed in blood culture vials in 34 samples and in Schaedler’s broth in 27 samples [18].

All isolates were identified by mass spectrometry (Biotyper on a Microflex LT mass spectrometer, Bruker Daltonics, Bremen, Germany) and their antimicrobial susceptibility tested by disk diffusion. Confirmation of the minimal inhibitory concentration (MIC) was performed by ellipsometry or broth microdilution (Etest, BioMérieux, Marcy l’Etoile, France or UMIC, Biocentric, Bandol, France) on available isolates and reinterpreted according to the EUCAST 2018 guidelines [19].

### Statistics

Pearson chi-square test or Fisher exact test were used to compare categorical variable. For all tests, *p* values ≤0.05 were considered as statistically significant.

Quantitative variables are presented as median and interquartile range (IQR), and qualitative variables are presented as number of occurrences and relative frequencies.

Statistical analysis was performed with Statistical Package for Social Science for Windows version 17.0 (SPSS, Chicago, USA).

## Results

**Fig. 3:**
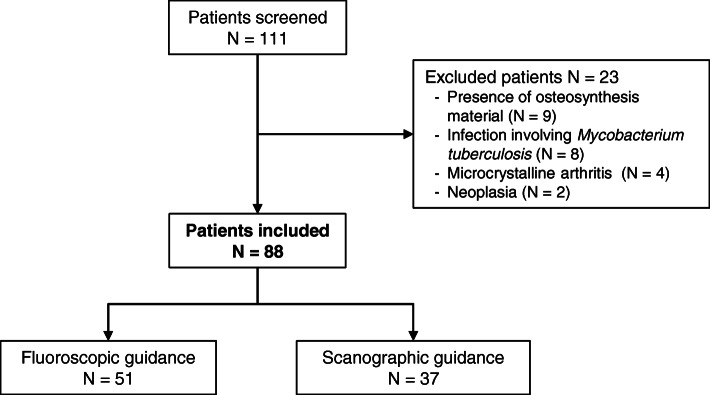
Flow chart

A total of 111 medical files were reviewed, and 88 patients with clinical and morphological diagnosis of PVO, assessed by the committee, were included (Fig. 3). Out of the 23 patients excluded, exclusion criteria were: presence of osteosynthesis material (*n* = 9), infection involving *Mycobacterium tuberculosis* (*n* = 8), microcrystalline arthritis (*n* = 4), and neoplasia (*n* = 2).

Median age was 67.0 years, and mean age was 61.7 years (range 20–89), with a sex ratio (M/F) of 2.7. Immunosuppression was present in 9 (10.2%) patients, and half of patients (*n* = 42; 47.7%) had acute PVO presentation. Previous antibiotic treatment was notified in 19 (21.6%) patients. Epidural abscess and paravertebral infiltration were reported respectively in 13 (18.3%) and 26 (36.1%). The main localization of PVO was lumbar (*n* = 51; 58.0%), and the main bacteria involved was staphylococci (*n* = 25; 47.3%).

Overall, 53 (60.2%) patients had positive DVB cultures, whereas 35 (39.8%) patients had negative DVB cultures (Table 1). Patients’ baseline characteristics according to the DVB results are presented in Table 1.

**Table 1:** Baseline characteristics of study patients according to the disco-vertebral biopsies (DVB) results

Patients	Positive DVB (***n*** = 53)	Negative DVB (***n*** = 35)	***P***-value
Age (years), median [IQR]	67.0 [54.0; 80.0]	63.0 [47.5; 76.0]	0.31
Sex ratio (M/F)	2.5	2.9	0.79
Immunosuppression, n (%)	5 (9.4)	4 (11.4)	0.76
Previous spine surgery, n (%)	8 (15.0)	2 (5.7)	0.19
**PVO presentation, n (%)**			
Acute	25 (47.2)	17 (48.6)	1.0
Subacute	6 (11.3)	7 (20.0)	0.26
Chronic	9 (17.0)	4 (11.4)	0.58
**Previous antibiotic therapy**, n (%)	9 (17.0)	10 (28.6)	0.2
**Fever,** n (%)	2 (3.8)	1 (2.9)	0.82
**Biology**, median [IQR]			
WBC count (10^9^/L)	9.5 [7.4; 11.5]	7.6 [6.2; 12.2]	0.44
CRP level (mg/L)	47.0 [26.0; 120.0]	26.5 [8.5;123.5]	0.42
ALP (UI/L)	117.0 [90.0; 179.5]	144.0 [100.0; 212.0]	0.24
**Biology**, n (%)			
WBC > 10 (10^9^/L)	19 (47.5)	10 (35.7)	0.33
CRP > 50 mg/L	19 (86.4)	12 (42.8)	0.77
CRP > 100 mg/L	14 (34.1)	8 (28.6)	0.62
ALP > 118 UI/L	20 (55.6)	18 (64.2)	0.48
**Radiology**, n (%)			
Epidural abscess	8 (19.5)	5 (16.7)	0.76
Psoas abscess	7 (17.1)	8 (26.7)	0.33
Discal abscess	11 (26.2)	8 (26.7)	0.96
Paravertebral abscess	12 (29.3)	9 (30.0)	0.95
Vertebral endplates erosions > 50%	16 (37.2)	9 (28.1)	0.41
Paravertebral infiltration ≥10 mm	22 (52.4)	4 (13.3)	**0.0006**
**Spine Level**, n (%)			
Cervical	3 (5.7)	2 (5.7)	1.0
Thoracic	20 (37.7)	12 (34.3)	0.7
Lumbar	30 (56.6)	21 (60.0)	0.8
**Microbiology**, n (%)			
Monomicrobial infection	41 (77.4)	NA	NA
Coagulase negative staphylococci	15 (28.3)	NA	NA
*Staphylococcus aureus*	10 (18.9)	NA	NA
*Cutibacterium acnes*	7 (13.2)	NA	NA
*Escherichia coli*	3 (5.7)	NA	NA
Streptococci	4 (7.5)	NA	NA
Anaerobes	3 (5.7)	NA	NA
*Pseudomonas aeruginosa*	2 (3.8)	NA	NA
Enterococci	1 (1.9)	NA	NA
*Klebsiella* spp.	1 (1.9)	NA	NA

*ALP* Alkaline phosphatase, *CRP* C-reactive protein, *DVB* Disco-vertebral biopsy, *IQR* Interquartile range, *NA* Not applicable, *NS* Not significant, *PVO* Pyogenic vertebral osteomyelitis, *WBC* White cell blood

There was no significant difference between the two groups concerning immunosuppression (*p* = 0.76), previous spine surgery (*p* = 0.19), antibiotics during the previous 21 days before DVB (*p* = 0.2), disease presentation (acute vs. subacute, *p* = 0.26; acute vs. chronic, *p* = 0.58), fever during the following 24 h DVB (*p* = 0.81). Regarding biologic data, we found no significant difference between the two groups. But, paravertebral thickening more than 10 mm (in cervical, thoracic and lumbar spine) was present in 22/53 (52.4%) cases with positive DVB vs. 4/35 (13.3%) cases with negative DVB which was significantly different (*p* < 0.001; odds ratio (OR) = 5.4, 95% Confidence interval (CI) [1.58–24.10]). Concerning the others imaging findings, no significant difference was found.

The most frequent causative micro-organisms isolated were coagulase negative Staphylococci (*n* = 15, 28.3%), and *Staphylococcus aureus* (*n* = 10, 18.9%).

DVB was performed under fluoroscopic guidance in 51/88 (58.0%) cases vs. 37/88 (42.0%) cases under scanographic guidance. DVB cultures were positive under fluoroscopic guidance in 34/51 cases (66.7%) vs. 19/37 cases (51.3%) under scanographic guidance (*p* = 0.15) (Table 2). Concerning the lumbar spine, positive rate of DVB cultures under fluoroscopic guidance was significantly different than that under scanographic guidance (25/36 (69.4%) vs. 5/15 (33.3%), *p* = 0.02, respectively) (Table 2). Concerning DVB performed in the cervical and thoracic levels, no significant difference was shown between fluoroscopic and scanographic guidance (Table 2).

**Table 2:** Positive rate of disco-vertebral biopsies (DVB) cultures according to modality of guidance and spine level

	Fluoroscopic Guidance	Scanographic guidance	***P-***value
**All spine levels**	34/51	19/37	0.15
**Cervical level**	3/4	0/1	0.4
**Thoracic level**	6/11	14/21	0.7
**Lumbar level**	25/36	5/15	0.02

There were no biopsy-related major complications such as hemorrhage, infection, fracture or major pneumothorax.

## Discussion

Our cohort study among patients with PVO with negative blood cultures who underwent DVB showed that DVB positive cultures are significantly associated with a paravertebral thickening more than 10 mm and DVB with fluoroscopic guidance at lumbar level. These results could help the physicians manage patients presenting an indication for DVB in case of PVO.

Our population is representative of the general population with PVO, with the same mean age according to a recent epidemiological study [20].

The most frequently identified microorganisms were coagulase negative Staphylococci, whereas in the literature, *Staphylococcus aureus* is the most frequently involved [8]. It should be noted that we only included patients with negative blood culture, which explains the large number of coagulase negative staphylococci PVO. Our PVO cases were mostly monomicrobial, as previously reported [21].

According to our results, age, sex, clinical or biological characteristics were not statistically associated with a positive DVB, as reported in literature [13, 14]. Moreover, in a retrospective study including 75 patients, Wu et al. did not show significant difference in the culture positivity rate with regard to fever > 38 °C, elevated WBC ≥10^9^/L, elevated C-Reactive Protein level ≥ 6 mg/L, as did we [17].

In our study, univariate analysis showed that paravertebral tissue ≥10 mm of thickening on axial MR scans is a predictive factor of microbiological positivity of DVB (Table 1). This result is in line with the retrospective study by Spira et al., which included only 34 patients who underwent DVB under scanographic guidance, and showed that a threshold of paravertebral soft tissue > 5 mm reliably indicated successful pathogen detection [22].

Considering type of guidance, DVB under fluoroscopic guidance at the lumbar level seems more sensitive to identify causative micro-organisms (Table 2).

It could be explained by the fact that fluoroscopic guidance allows a wide range of craniocaudal angulation, an easy perception of the needle position in the vertebral body in the lateral view, a short procedure time, and real-time visualization and control of the needle position [23]. Regarding the L5-S1 space, fluoroscopic guidance is almost systematic because the disk is very recessed. Indeed, DVB at this level need a larger craniocaudal angulation, which is impossible under scanographic guidance.

Nevertheless, no significant difference between fluoroscopic guidance and scanographic guidance had been shown at other level, which could be due to the small sample size [14, 17, 24].

This original finding was not found in a previous meta-analysis, which did not show any statistically significant difference between fluoroscopic and scanographic guidance of DVB concerning their accuracy [25]. Our results could be due to the expertise of our hospital, which is a referral center for bone and joint infections. Moreover, the high rate of positive microbiological diagnosis in our cohort is probably due to the microbiological analysis procedure of the sample which is optimal (Table 3) [8, 15, 16].

**Table 3:** Meta-analysis concerning the diagnostic yield of disco-vertebral biopsy for pyogenic vertebral osteomyelitis

References	Number of patients included	Type of study		Study of predictive positive factors of DVB	% of positive DVB
Osenbach 1990 [26]	40	Retrospective	**Multicentric**	No	90% (36/40)
Bontoux 1992 [27]	82	Retrospective	Monocentric	No	47.5% (19/40)
Perronne 1994 [28]	39	Retrospective	Monocentric	No	74% (29/39)
Torda 1995 [29]	15	Retrospective	Monocentric	No	73% (11/15)
Fouquet 1996 [30]	67	Retrospective	Monocentric	No	51% (34/67)
Rieneck 1996 [31]	14	Retrospective	Monocentric	No	57% (8/14)
Honan 1996 [32]	12	Retrospective	Monocentric	No	83% (10/12)
Carragee 1997 [33]	44	Retrospective	Monocentric	No	61% (27/44)
Jensen 1998 [34]	133	Retrospective	**Multicentric**	No	40% (53/133)
Hadjipavlou 2000 [35]	21	**Prospective**	Monocentric	No	57% (12/21)
Lucio 2000 [25]	20	Retrospective	Monocentric	No	75% (15/20)
Chew 2001 [3]	43	Retrospective	**Multicentric**	No	91% (39/43)
Ben Taarit 2002 [36]	21	Retrospective	Monocentric	No	48% (10/21)
Mc Henry 2002 [7]	253^a^	Retrospective	**Multicentric**	No	69% (86/124)
Nolla 2002 [37]	21	Retrospective	Monocentric	No	52% (11/21)
Cherasse 2003 [38]	35	Retrospective	Monocentric	No	69% (24/35)
Rankine 2004 [24]	20	Retrospective	Monocentric	No	40% (8/20)
Mylona 2008 [22]	NA	NA	NA	No	79%
D’agostino 2010 [39]	81	Retrospective	Monocentric	No	76% (62/81)
Rio 2014 [40]	58	Retrospective	Monocentric	No	64% (37/58)
Gras 2014 [13]	136	Retrospective	**Multicentric**	Yes	43% (59/136)

*DVB* Disco-vertebral biopsy, *NA* Not available

^a^Among the 253 included patients, 124 patients received PVB

No biopsy-related complications were recorded.

One could deplore a lack of information concerning the quality of the biopsy specimen such as the number of samples or the size of the needle used to perform DVB or aspiration of purulent fluid during DVB, which is limited due to the retrospective design. However, several authors did not show any significant difference concerning the needle type and his diameter in terms of diagnostic rate [8, 12, 15, 23].

Limitations of the study include its retrospective study design, which may have resulted in missing key variables; there is possibility of selection bias. In addition, our sample size may not be large enough to detect other significant predictive factors of positivity. But, PVO is a rare condition, so it is difficult to perform a prospective study with a large sample of patients and the same DVB technique.

## Conclusion

Fluoroscopic guidance of DVB seems to be better than scanographic guidance at the lumbar spine level. It is an accurate method for identifying the microorganism involved in PVO, in order to properly manage this disease. It is a safe and well-tolerated procedure. We demonstrated that a paravertebral thickening ≥10 mm is a predictive positive factor for DVB.

## Data Availability

The datasets used and/or analysed during the current study are available from the corresponding author on reasonable request.

## Abbreviations

CI: Confidence interval
DVB: Disco-vertebral biopsy
IQR: Interquartile range
MIC: Minimal inhibitory concentration
MRI: Magnetic resonance imaging
OR: Odds ratio
PVO: Pyogenic vertebral osteomyelitis
